# Characterisation of major histocompatibility complex class IIa haplotypes in an island sheep population

**DOI:** 10.1007/s00251-019-01109-w

**Published:** 2019-02-22

**Authors:** Kara L. Dicks, Josephine M. Pemberton, Keith T. Ballingall

**Affiliations:** 1 Institute of Evolutionary Biology, School of Biological Sciences, University of Edinburgh, Edinburgh, EH9 3FL UK; 2 Moredun Research Institute, Pentlands Science Park, Bush Loan, Penicuik, Midlothian, EH26 OPZ UK

**Keywords:** Major histocompatibility complex, Soay sheep, Haplotype, Balancing selection

## Abstract

The ovine MHC class IIa is known to consist of six to eight loci located in close proximity on chromosome 20, forming haplotypes that are typically inherited without recombination. Here, we characterise the class IIa haplotypes within the Soay sheep (*Ovis aries*) on St. Kilda to assess the diversity present within this unmanaged island population. We used a stepwise sequence-based genotyping strategy to identify alleles at seven polymorphic MHC class IIa loci in a sample of 118 Soay sheep from four cohorts spanning 15 years of the long-term study on St. Kilda. *DRB1*, the most polymorphic MHC class II locus, was characterised first in all 118 sheep and identified six alleles. Using *DRB1* homozygous animals, the *DQA* (*DQA1*, *DQA2* and *DQA2-like*) and *DQB* (*DQB1*, *DQB2* and *DQB2-like*) loci were sequenced, revealing eight haplotypes. Both *DQ1*/*DQ2* and *DQ2/DQ2-like* haplotype configurations were identified and a single haplotype carrying three *DQB* alleles. A test sample of 94 further individuals typed at the *DRB1* and *DQA* loci found no exceptions to the eight identified haplotypes and a haplotype homozygosity of 21.3%. We found evidence of historic positive selection at *DRB1*, *DQA* and *DQB*. The limited variation at MHC class IIa loci in Soay sheep enabled haplotype characterisation but showed that no single locus could capture the full extent of the expressed variation in the region.

**Electronic supplementary material:**

The online version of this article (10.1007/s00251-019-01109-w) contains supplementary material, which is available to authorized users.

## Introduction

The major histocompatibility complex (MHC) is a genomic region containing highly polymorphic genes which encode cell surface proteins involved in the presentation of pathogen-derived peptides to T cells, enabling an immune response (Klein 1986). The highly complex and polymorphic nature of the MHC region has made it a focus of many studies in immunology and evolution; however, these features make it difficult to develop locus-specific assays to genotype individual loci. Over evolutionary time, mammalian MHC loci can be viewed as going through a birth and death process (Nei et al. 1997). New MHC loci are thought to be created through gene duplication events, resulting in multiple loci harbouring similar alleles. Some are lost through decay, producing pseudogenes and gene fragments, which are characteristic of mammalian MHC regions. Additionally, allelic diversity within many MHC loci is high, and selection may favour the maintenance of numerous and divergent alleles within a population (Wakeland et al. 1990; Lenz 2011). Genotyping the MHC region is therefore often particularly challenging, and locus-specific assays can difficult to develop, as multiple loci and pseudogenes may co-amplify with primers that are too generic, whilst allelic dropout may occur with primers that are too specific (Babik et al. 2009).

The MHC class II loci exhibit high linkage disequilibrium (LD) (Lee et al. 2012), and many studies take advantage of this by genotyping a single locus, assuming that it is representative of the full haplotypic diversity (e.g. Babik et al. 2005; Harf and Sommer 2005; Bollmer et al. 2007; Biedrzycka et al. 2011; Gelasakis et al. 2013; Kamath et al. 2014). However, despite high LD, selection favouring particular alleles could mean that some alleles at specific loci are identical across different haplotypes (de Bakker et al. 2006; Traherne et al. 2006). For example, in sheep, an allele at one locus can be found in combination with multiple alleles at other loci (Hickford et al. 2007; Ballingall et al. 2015; Ali et al. 2016). Genotyping only a single locus may disguise variation at other loci, reducing the power to detect differing selection pressures. This highlights the need to characterise haplotypic variation when studying the MHC.

The unmanaged but intensively studied Soay sheep (*O. aries*) population on the island of Hirta, in the St. Kilda archipelago, Scotland, presents an excellent opportunity to study MHC selection in a large mammal. The Hirta population of Soay sheep originated from 107 animals which were translocated from the neighbouring smaller island of Soay in 1932 (Clutton-Brock et al. 2004), and the population has remained closed ever since. MHC variation within the study population was therefore expected to be limited compared with larger populations experiencing immigration. Since 1985, the long-term study of the Soay sheep has collected DNA samples, as well as life history and phenotype data, for many thousands of individuals (Clutton-Brock et al. 2004). Fully characterising the MHC class IIa haplotype variation within the Soay population might, therefore, be feasible.

The two major families of antigen-presenting MHC molecules are class I, which present endogenous antigens (primarily from intracellular pathogens) to CD8+ T cells, and class II which present exogenous antigens (primarily from extracellular pathogens) to CD4+ T cells (Klein 1986). Unusually, the class II region within ruminants is split into two distinct subregions, class IIa and IIb (Andersson et al. 1988; van Eijk et al. 1995), with the classical class II loci, which have previously been associated with parasite resistance (see Lee et al. 2011), clustered in the class IIa region. The class IIa loci include the highly polymorphic *DRB1*, *DQA* and *DQB* loci, as well as the less polymorphic *DRA* (Ballingall et al. 2010). Duplicated pairs of *DQA* and *DQB* loci have been identified in domestic sheep, *Ovis aries* (Scott et al. 1987; Wright and Ballingall 1994; Ballingall et al. 2015, 2018b). Three types of *DQA* alleles (*DQA1*, *DQA2* and *DQA2-like*; Ballingall et al. 2015) and *DQB* alleles (*DQB1*, *DQB2* and *DQB2*-*like*; Ballingall et al. 2018a, b) have been identified. *DQA1* and *DQA2* are known to be different loci, as are *DQB1* and *DQB2*; however, the origins of the *DQA2*-*like* and *DQB2*-*like* alleles are less well defined. Typically, *DQA2*-*like* and *DQB2-like* alleles are found on haplotypes in conjunction with *DQA2* and *DQB2*, and the *DQA1* and *DQB1* loci are absent. Therefore, the two typical haplotype configurations are *DQA1 + DQA2* with *DQB1 + DQB2* and *DQA2* + *DQA2-like* with *DQB2* + *DQB2-like*. Whether the *DQA2-like* and *DQB2-like* alleles represent independent loci or are simply divergent alleles at the *DQA1* and *DQB1* loci remains unclear (Ballingall et al. 2015, 2018b). A recent study by Ali et al. (2016) identified haplotypes with all three allele types, which would suggest that the *DQA2*-*like* and *DQB2-like* alleles are derived from independent loci.

A previous study of MHC variation in Soay sheep using the OLADRB microsatellite located in the second intron of the class IIa locus *DRB1* found evidence for selection acting on this region (Paterson et al. 1998). However, as outlined above, how well the single OLADRB microsatellite locus represents diversity across the MHC class IIa region of the Soay sheep is unknown. Since the Paterson et al. (1998) study, single locus genotyping methods targeting the polymorphic regions of the classical class IIa loci have been developed for domestic sheep. These include *DRB1* (Ballingall and Tassi 2010), *DQA* (Ballingall et al. 2015) and *DQB* (Ballingall et al. 2018b). In this study, we aim to (1) genotype a sample of Soay sheep at the classical *DRB1* and *DQ* loci using sequence-based genotyping and (2) define the MHC class IIa haplotypes in the Soay sheep study population using animals identified as homozygous for each of the *DRB1* alleles. Additionally, we aim to (3) look for evidence of positive selection acting on ovine MHC alleles during their evolutionary history. Characterising the MHC class IIa loci in this population will facilitate the development of a method to determine haplotypes for large numbers of individuals, enabling subsequent investigation of the evolutionary mechanism maintaining diversity within this region.

## Methods

### Study system

Monitoring of Soay sheep in the Village Bay area on Hirta has been carried out intensively since 1985 (Clutton-Brock et al. 2004), including catching lambs in spring for weighing, ear-tagging and sampling for genetic analysis. Most sheep are also caught in August, when phenotypic measurements, faecal samples for strongyle egg counts and blood samples are taken. During the August catches of 2012 to 2014, aliquots of blood were collected into Tempus™ Blood RNA Tubes (ThermoFisher Scientific).

### Genomic DNA preparation

Genomic DNA (gDNA) was previously extracted from either peripheral blood or ear punch tissues using either the phenol/chloroform method (Bancroft et al. 1995) or QIAGEN DNeasy or QIAamp DNA Mini kits (QIAGEN, Dusseldorf, Germany) following the manufacturer’s protocol.

### Sequence-based genotyping

#### DRB1 genotyping

The *DRB1* locus is the best characterised MHC class II locus in *O. aries*, and the Immuno Polymorphism Database (IPD-MHC - https://www.ebi.ac.uk/ipd/mhc) contains over 100 ovine *DRB1* alleles and corresponding allelic nomenclature. Locus-specific primers and a sequence based genotyping method, which targets the polymorphic second exon, have previously been developed (Ballingall and Tassi 2010). The primer pair 330_F and 329_R (Ballingall and Tassi 2010) was tested initially as it generates full *DRB1* exon 2 sequences (Table 1). However, a 1-bp deletion in the DRB1*13:01 allele generated a mixed sequence from which the alleles could not be unambiguously identified. Therefore, the forward primer 455_F, a modification described in Corbishley et al. (2016) which sits downstream of the deletion, was used in preference (Table 1). Between 27 and 31 sheep were randomly selected from each of four cohorts (1993, 1998, 2003, 2008) and genotyped to identify *DRB1* homozygous individuals for subsequent analysis of *DQ* diversity, with the expectation that *DRB1* homozygotes were more likely to be homozygous at *DQ* loci due to linkage disequilibrium.

**Table 1:** Details of the primers used and their PCR conditions

Target loci	DNA target	Primer name	Primer sequence	T_A_	PCR cycle times (s)	Primer reference
*DRB1*	gDNA	330_F	ATTAGCCTCYCCCCAGGAGKC	55	30, 30, 30	(Ballingall and Tassi 2010)
		455_F	TATCCCGTCTCTGCAGCACATTTC	58		(Corbishley et al. 2016)
		329_R	CACCCCCGCGCTCACCTCGCCGC	55–58		(Ballingall and Tassi 2010)
*DQA1*	gDNA	*DQA1*_F	ACCTGACTCACCTGACCACA	55	60, 60, 60	(Ballingall et al. 2015)
		*DQA1*_R	AACACATACTGTTGGTAGCAGCA	55		
*DQA2* and *2-like*	gDNA	*DQA2*_F	ACTACCAATCTCATGGTCCCTCT	58		
		*DQA2*_R	GGAGTAGAATGGTGGACACTTACC	58		
*DQA1* and *2*	cDNA	244_F	GCTGAGMCCACCTTGAGAASAG	55	60, 60, 60	(Ballingall et al. 2015)
		241_R	TGAGATGATACAGCATCTTAAGTCC	55		
*DQA1*, 2 and 2-like	cDNA	348_F	GAGGATGGTCCTGAACAGAGC	55		
		357_R	GAGGAGGGCAGAAGAAGAAAA	55		
*DQB*	gDNA	*DQB*_F	CCCCGCAGAGGATTTCSTG	58–60	30, 30, 30	(Ballingall et al. 2018b)
*DQB1*	gDNA	DQB1_R	CGGCACTCACCTCGCCGCTGC	60		
*DQB2* and *2-like*	gDNA	DQB2_R	ACGCTCACCTCGCCGCTGCC	58		
*DQB1* and *2*	cDNA	245_F	TGGGTGTTGACTACCATTAST	55	60, 60, 60	(Ballingall et al. 2018b)
		248_F	ACGCASSYATTAYAGAAGAGC	55		
*DQB2-like*	cDNA	392_F	ATTAGTTGTTCCTTTTTTCTC	55		
		395_R	AAAATATCCTCAGGAGTCAGC	55		
		401_R	CAAGAACACGCAGCTATTACA	55		

#### DQA genotyping

For each *DRB1* allele identified, four *DRB1* homozygous individuals were genotyped at the *DQA* loci (*DQA1*, *DQA2* and *DQA2-like*). Individuals homozygous for the rare allele DRB1*10:01 were not identified from the initial *DRB1* screen, and thus, *DQA* haplotypes were determined from heterozygous individuals. Primers *DQA1*_F and *DQA1*_R were used to amplify *DQA1* and primers *DQA2*_F and *DQA2*_R were used to amplify *DQA2* and *DQA2-like* (Table 1). For *DQA2/DQA2-like* homozygous individuals, the *DQA1* primers were not expected to generate a PCR product and the *DQA2* primers were expected to amplify both *DQA2* and *DQA2-like* loci and therefore generate a product that was heterozygous in appearance.

#### DQB genotyping

*DQB* loci (*DQB1*, *DQB2* and *DQB2-like*) were characterised in individuals which were both *DRB1* and *DQA* homozygous. Primers *DQB*-F and *DQB1*-R or *DQB2*-R were used to amplify the *DQB* loci (Table 1). The *DQB* primers were not completely locus-specific and some cross-amplification was expected, depending upon the alleles present (Ballingall et al. 2018b). Due to the cross-amplification of loci, it was not possible to unambiguously identify *DQB* alleles from DRB1*10:01 heterozygotes. A subsequent screen of a much larger number of individuals (data not included here) was necessary to find individuals homozygous for the DRB1*10:01 allele, and the corresponding *DQB* alleles were identified from these homozygotes.

#### PCR amplification

PCR reactions were carried out in a final volume of 25 μL and contained 12.5 μL Promega GoTaq Green mastermix, 0.5 μM of each primer, approximately 25 ng genomic DNA and water. Cycling conditions were 94 °C for 5 min, then 35 cycles of 94 °C for 30 or 60 s, 55–58 °C for 30 or 60 s and 72 °C for 30 or 60 s (see Table 1) and a final extension of 72 °C for 5 min. PCR products were checked using gel electrophoresis on a 1% agarose gel.

#### RT-PCR amplification of full length DQ transcripts

Full-length transcripts were amplified from RNA to validate the previously unidentified sequences from genomic DNA. For individuals for which Tempus™ Blood RNA Tubes were available (see Study system), *DRB1* and *DQA* loci were amplified from genomic DNA to identify homozygous individuals for RNA extraction. Total RNA was extracted from 3 mL of Tempus tube blood stored at − 20 °C using the Tempus™ Spin RNA Isolation Kit (ThermoFisher Scientific) at half the recommended volumes. Reverse Transcription was carried out using the ImProm-II™ kit (Promega) with oligo(dT)_15_ primers. RT-PCR reactions were carried out using the primer combinations described in Table 1. No single primer set amplified all alleles. As RNA was not available for any individuals homozygous for haplotype H, F/H heterozygous individuals were used instead.

#### Cloning of multi-allelic PCR products

In order to phase novel alleles from heterozygous or multi-locus amplifications, PCR fragments were cloned into the pGEM-T easy vector (Promega). The presence of the correct insert was confirmed using colony PCR and plasmid DNA from 12 to 20 colonies was purified for sequencing using QIAprep Spin Miniprep Kit (Qiagen).

#### Sequencing and sequence analysis

PCR products were purified using exonuclease I and Antarctic phosphatase, except *DQB2* PCR products which were gel-purified using the Macherey-Nagel Nucleospin Gel and PCR Clean Up kit, prior to Sanger sequencing. Alleles were sequenced using BigDye 3.1 chemistries on an AB 3730 genetic analyser.

Sequence analysis was carried out in Geneious 7.1.9. For *DRB1* sequences, heterozygous peaks were called using the Heterozygote Plugin and checked by eye. *DRB1* sequences were then compared to alleles from the IPD-MHC database using a custom BLAST. *DRB1* genotypes were called when known alleles accounted for all variants within the sequence.

*DQA* and *DQB* sequences were also analysed using the Heterozygote Plugin within Geneious 7.1.9 to call heterozygous peaks. Due to multi-locus amplification, some sites contained three or four peaks, which were called by eye. A custom BLAST was used to compare sequences to an appropriate *DQA* database (Ballingall et al. 2015) or *DQB* database (Ballingall et al. 2018b). Genotypes were only called when all variants were accounted for by known alleles. Following cloning and RT-PCR of unknown alleles, the custom BLAST was updated to include the new alleles, and uncalled sequences were then compared to the updated database.

#### Nomenclature

The novel full-length *DQA* and *DQB* alleles identified here were named according to the nomenclature system described in Ballingall et al. (2015, 2018), were submitted to the European Nucleotide Archive (ENA; accession numbers LR025203-LR025213) and were included in the IPD-MHC database (Ballingall et al. 2018a). Genomic fragments that were considered too short to receive official nomenclature, i.e. those for which full-length transcripts could not be obtained, fell into one of two categories. Either, they matched an existing allele in GenBank that is also too short to receive official nomenclature, in which case, they were named after the accession number of the existing allele. Alternatively, the allele does not have a match in GenBank and was named according to the locus and haplotype designation and submitted to the ENA (accession numbers LR025788–LR025790).

### Phylogeny of *DQB*

Due to the lack of locus-specific *DQB* primers, loci could not be determined for all amplified alleles, and thus, a phylogeny of the *DQB* sequences was generated to help clarify their *DQB* locus of origin. The *DQB* alleles sequenced were aligned using Clustal Omega (Sievers et al. 2011), along with previously identified *DQB* sequences from *O. aries* (Ballingall et al. 2018b), with domestic pig (accession NM_001113694.1.1) and human (accession M24364.1) *DQB* sequences as outgroups. Model selection for MrBayes, implemented in Topali v2 (Milne et al. 2009), selected the K80 model of DNA substitution (Kimura 1980) with gamma distribution. The phylogeny was generated in Geneious v7.1.9 using MrBayes 3.2.6 (Huelsenbeck and Ronquist 2001) using 1,000,000 generations, with a burn-in of 25,000 generations.

### Validation of haplotypes

To confirm that the haplotypes characterised in homozygous individuals were consistent, *DRB1* and *DQA* loci were genotyped in an additional 95 sheep previously not genotyped by this study. These sheep were not first order relatives of each other and were born between 1984 and 2010. These individuals were selected as they were genotyped on the Ovine Infinium HD SNP BeadChip for a previous study (Johnston et al. 2016). These individuals represent the maximum genetic variation within the population for the given number of individuals (Johnston et al. 2016).

### Positive selection analyses

Positive selection acting over the evolutionary history of MHC alleles can be detected by comparing the ratio of non-synonymous (dN) to synonymous (dS) substitutions across sites (dN/dS) (Hill and Hastie 1987; Hughes and Nei 1988) and was assessed using CODEML implemented within the PAML4 package (Yang 2007). Analyses were carried out for *DRB1* and for the duplicated *DQ* loci as all *DQA* loci combined (*DQA1*, *DQA2*, *DQA2-like*) or all *DQB* loci combined (*DQB1*, *DQB2*, *DQB2-like*) in order to maximise allelic sample sizes. As selection may differ between the duplicated loci, analyses were carried out separately for *DQA1*, *DQA2*, *DQB1* and *DQB2*, although not for *DQA2-like* and *DQB2-like* because allele sample sizes were too small (*n* = 3 and *n* = 4, respectively). Alleles included in positive selection analyses are listed in Supplementary Table 2.

CodeML input neighbour-joining trees for each locus as described above were produced in Geneious v7.1.9 using HKY model and 5000 bootstrap replicates using *O. aries* alleles with assigned nomenclature (https://www.ebi.ac.uk/ipd/mhc; Ballingall et al. 2011, 2018a). The null hypothesis that neutrality is operating was first modelled for each locus using M1a in PAML, which assumes two possible classes of selection, *ω*_0_ < 1 as estimated from the data and *ω*_1_ = 1. This was then compared to the alternative hypothesis using model M2a in which positive selection is assessed using the two site classes considered under M1a as well as a third, *ω*_2_ > 1 as estimated from the data. The model which best fit the data was inferred by calculating the likelihood ratio test (LRT) *p* value calculated as two times the difference between the models, compared to the chi-squared distribution with two degrees of freedom (Yang 2007; Posada 2009). Type I errors can be inflated when using the LRT with high levels of recombination, so the models M7 and M8 were also tested in PAML as they have previously been shown to be more robust to recombination (Anisimova et al. 2003). Model M7 is the null model of neutrality with a *β* distribution, and M8 as the alternative hypothesis, with *β* and *ω*. Codon frequency model 2 (F3x4) was used throughout. Additionally, positive site selection analyses were carried out within CodeML (Bayes Empirical Bayes (BEB) within model M2a) and within HyPhy using FEL and MEME methods.

## Results

### Sequence-based genotyping

#### DRB1

Six *DRB1* alleles were identified among the 118 individuals from the four cohorts born in 1993, 1998, 2003 and 2008. All six alleles were represented in the IPD-MHC database and each allele was assigned to an individual haplotype (A–F; Table 2). Allele frequencies are shown in Supplementary Fig. 1. In total, 33 (27.5%) *DRB1* homozygous sheep were identified, with five of the six *DRB1* alleles represented by a minimum of three homozygous individuals. Only DRB1*10:01 was not represented in homozygote form within the 118 sheep tested.

**Table 2:** MHC class IIa haplotypes identified in the Soay sheep across DRB1, DQA and DQB loci. The lack of detection of an allele at a locus is indicted by a dash. An asterisk preceding the allele name indicates nomenclature follows IPD guidelines (Marsh et al. 2010). Novel alleles identified by this study are highlighted in italics and accession numbers are shown in brackets

Haplotype	DRB1	DQA1	DQA2	DQA2-like	DQB1	DQB2	DQB2-like
A	*01:01	–	*10:01:01 (LR025213)	*03:01:01 (LR025210)	*–*	*09:01:01 (LR025203)	*03:01:01 (LR025207)
B	*01:02	*03:01:01	*01:01:01	–	*02:01:01	*04:01:01	–
C	*03:02	Z28420	*07:01:01	–	LN868258	AJ238945	–
D	*10:01	*03:01:01	*02:01:01	–	*07:01:01 or *07:02:01^a^	*SoayB2-D (LR025789)*	–
E	*13:01	–	*01:02:01	*01:01:01	–	*10:01:01 (LR025204)	*01:01:01
F	*22:01	*03:02:01 (LR025209)	*09:01:02 (LR025211)	–	AJ23941^b^	*SoayB2-F* ^b^ *(LR025790)*	–
G	*01:01	–	*01:02:01	*01:01:01	–	*09:01:01 and *12:01:01 (LR025203) and (LR025205)	*01:01:01
H	*22:01	*04:02:01 (LR025208)	*04:02:01 (LR025212)	–	*NewB1-H (LR025788)*	*11:01:01 (LR025206)	–

^a^Only exon 2 could be amplified from haplotype D. Alleles DQB1*07:01 and DQB1*07:02 are identical throughout exon 2 and thus the precise allele designation cannot be achieved without longer transcripts

^b^Locus designation of alleles determined only by phylogenetic analysis—see Fig. 1

#### DQA loci

*DQA* genotyping of *DRB1* homozygous individuals and DRB1*10:01 heterozygotes provided evidence for an additional two haplotypes (G and H) (Table 2). The DRB1*01:01 and DRB1*22:01 alleles were each associated with two different *DQA* haplotypes. *DQA1* primers failed to amplify a product in three haplotypes, (*DQA1* null haplotypes, A, E and G), and a *DQA2-like* allele was identified in each of these haplotypes.

Six *DQA* alleles did not match full length sequences described in (Ballingall et al. 2015), and only one of these (DQA1*Z28420 on haplotype C) matched a sequence on GenBank (accession number Z28420). Full-length transcripts (768 bp) were generated for six of these *DQA* alleles (Table 2). *DQA1* could not be amplified from cDNA for haplotype C and official nomenclature could not be assigned, and instead, temporary nomenclature reflects the GenBank accession number (*DQA1-*Z28420*)*. Nevertheless, full exon 2 sequences were obtained for all alleles, except the final 12 bp of *DQA1*-Z28420 (Supplementary Fig. 2).

#### DQB loci

Both gDNA and cDNA *DQB* primer pairs exhibited varying degrees of cross-locus amplification, dependent upon the alleles present (Supplementary Table 1). The *DQB2-like* cDNA primers did not amplify any alleles from haplotypes known to carry a *DQB2-like* allele, likely due to polymorphisms at the primer binding regions but perhaps due to poor quality RNA samples.

A single allele at each of the loci matched alleles in Ballingall et al. (2018b), and a further two alleles at each of *DQB1* and *DQB2* matched alleles in GenBank (Table 2). The remaining alleles were novel and were assigned temporary nomenclature (Table 2). Full-length sequences for the two novel alleles on haplotypes E and H could not be generated, and full-length transcripts failed to amplify for the other five alleles in haplotypes C, D, F and H as shown in Supplementary Table 1. Three *DQB* alleles were identified from haplotype G, two from gDNA only and one from cDNA only (Supplementary Table 1). Full exon 2 sequences were obtained for all *DQB* alleles (Supplementary Fig. 3).

### Phylogeny of *DQB* loci

**Fig. 1:**
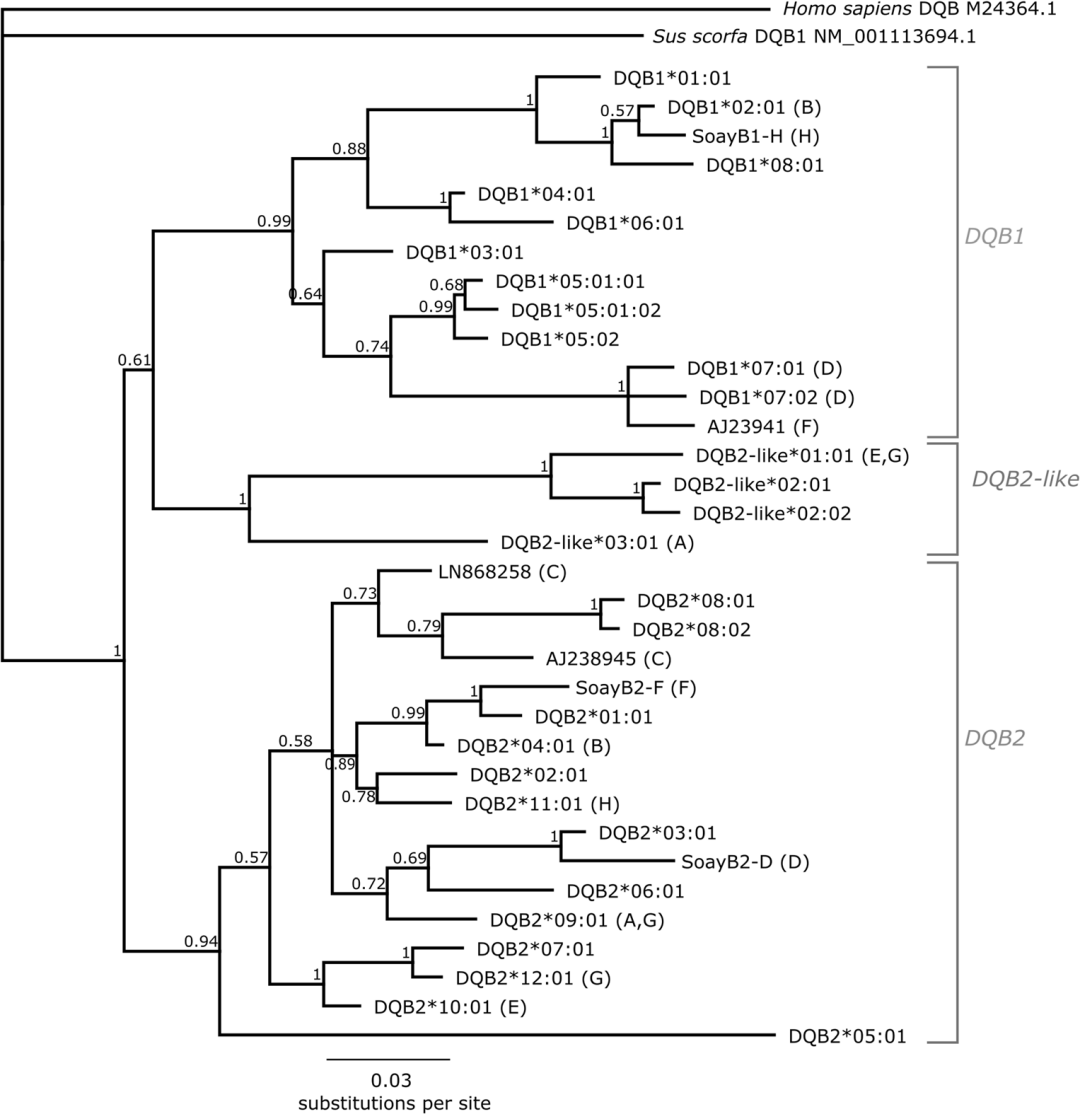
Phylogeny of DQB sequences estimated using MrBayes in Geneious. Letters in brackets indicate the Soay sheep haplotype that the allele was identified on. Numbers at branches are posterior probabilities

Phylogenetic analysis of the *DQB* loci revealed three distinct clusters, corresponding to the three loci *DQB1*, *DQB2* and *DQB2-like* (Fig. 1). All Soay *DQB* alleles are located within one of the three clusters. Alleles from each haplotype fell into two different clusters, with the exceptions that haplotype G carried two alleles that clustered within *DQB2*, and a third that clustered most closely with *DQB2-like*. Haplotype C alleles were both located within the DQB2 cluster; however, it should be noted that both alleles only include exon 2 and not the 3′ UTR where the phylogenetic signal is strongest. Haplotype C alleles were therefore designated according to the primer set with which they were amplified.

### Validation of haplotypes

Sequence-based genotyping across *DRB1*, *DQA* and *DQB* loci revealed eight haplotypes (Table 2). Direct sequencing of *DRB1* and *DQA* loci in 94 individuals did not reveal any deviations from the haplotypes identified using homozygous individuals. With the addition of the novel alleles on haplotypes C, F and H to the custom BLAST, alleles from all direct sequencing products could be determined, even from *DQA2*/*DQA2-like* heterozygous products from which four alleles co-amplified. Within these 94 individuals, 21.3% were homozygous.

### Positive selection and recombination analyses

**Fig. 2:**
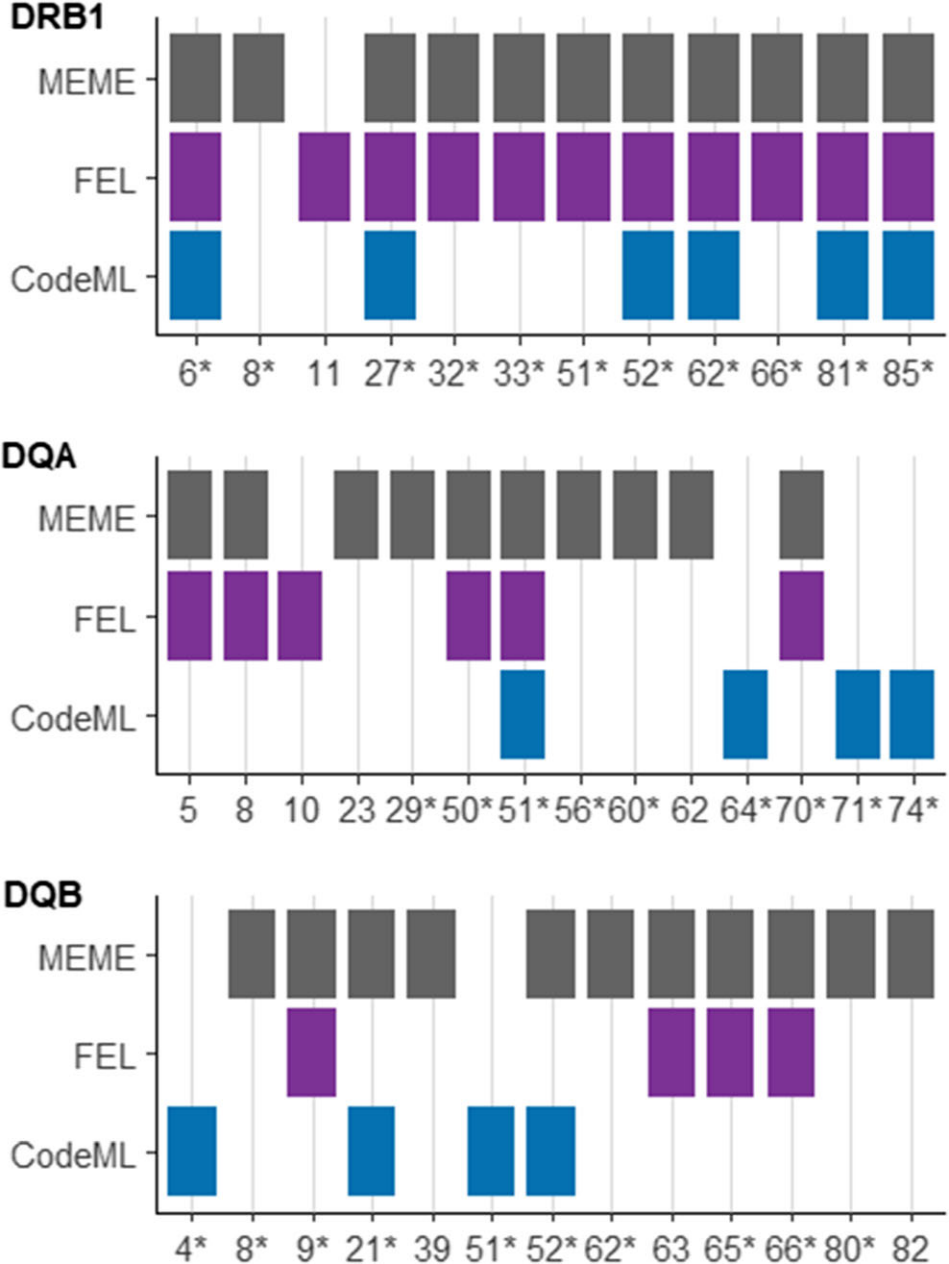
PSS for exon 2 of *DRB1*, *DQA* and *DQB* identified using BEB in CodeML, and FEL and MEME in HyPhy, where a coloured block indicates the codon was significant at > 0.95 probability (CodeML) or *p* < 0.05 (HyPhy). Sites denoted by asterisk indicate this position was identified as an antigen biding site within the human orthologue (Brown et al. 1993; Stern and Wiley 1994; Reche and Reinherz 2003; Bondinas et al. 2007)

For all loci, the null model of neutrality was rejected and the models incorporating positive selection were selected (Supplementary Table 3). Models M2a and M8 (positive selection) both fit the data significantly better than M1a and M7 (neutrality). However, after applying Bonferroni correction for multiple tests at *DQA* and *DQB*, (α-threshold becomes 0.017), the M1a and M7 models of neutrality are a better fit at *DQA2*. Additionally, positively selected sites were identified at all loci (Fig. 2), with 12 sites identified at *DRB1*, 14 at *DQA* and 13 at *DQB*, although only 10, 5 and 6 sites, respectively, were detected by more than one method. The majority of PSS at all three regions, *DRB1*, *DQA* and *DQB*, have also been identified as antigen binding sites in human homologues. Results of separate PSS analyses for *DQA1* and *DQA2*, as well as *DQB1* and *DQB2*, are shown in Supplementary Fig. 4 and found fewer PSS than when loci were combined with fewer overlapping PSS at *DQA1/DQA2* than for *DQB1/DQB2*.

## Discussion

Six *DRB1* alleles in Soay sheep were identified, which matched sequences previously identified in commercial Scottish sheep breeds and held in the IPD-MHC database. The level of homozygosity at the *DRB1* locus was 27.1% of 118 individuals, which is higher than previously observed in other breeds of sheep (Stear et al. 2005; Herrmann-Hoesing et al. 2008). However, at the MHC class IIa haplotype level, this is an over estimate of homozygosity as some individuals homozygous for DRB1*01:01 or DRB1*22:01 are heterozygous at *DQA* and *DQB* loci. The level of MHC class IIa haplotype homozygosity reduced to 21.3% in the 94 validation samples genotyped across the *DRB1* and *DQ* loci. Note that these samples were originally selected to maximise representation of Soay sheep diversity, so it is possible they are not totally representative of the population as a whole. No dramatic change in *DRB1* allele frequencies was observed over time (Supplementary Fig. 1), but sample sizes were too small to analyse temporal trends statistically. Further investigation at the population level will provide better estimates of the level of homozygosity and whether haplotype frequencies have changed over time within the Soay sheep population.

*DQA* and *DQB* genotyping revealed a total of eight MHC class IIa haplotypes in the Soay sheep. *DRB1* and *DQA* genotyping from MHC class IIa homozygous animals was relatively straightforward due to locus-specific primers (although the *DQA2* primer pair did co-amplify *DQA2*-*like* alleles). *DQB* primer pairs, however, showed varying levels of cross-amplification depending upon the alleles carried by the haplotype, which made phasing of alleles challenging without extensive cloning. The limited amount of MHC class IIa haplotype diversity has made identifying variation at the *DQA* and *DQB* loci from homozygous animals possible. Furthermore, assigning *DQB* alleles to loci was only possible using phylogenetic analysis. *DQB* genotyping would be improved by further development of locus-specific primers, although this is challenging due to limited genomic sequence in this region and the lack of locus-specific characteristics in exon 2 (van Oorschot et al. 1994; Wright and Ballingall 1994). Both known *DQ* haplotype configurations (*DQ1 + DQ2* and *DQ2 + DQ2-like*) were identified, as well as a novel configuration (two *DQB2* alleles with *DQA2 + DQA2-like + DQB2-like* on haplotype G) in Soay sheep. Both *DQB2* alleles on haplotype G were phylogenetically clustered within the *DQB2* group (Fig. 1) and feature *DQB2* locus-specific 3′ UTR sequences. The two *DQB2* alleles were amplified from either cDNA (DQB2*12:01) or gDNA (DQB2*09:01). Whilst this might suggest that the DQB2*09:01 allele is not transcribed, many of the *DQB* alleles identified from Soay sheep failed to amplify from cDNA, including both *DQB2-like* alleles. Previous work has shown that all DQA and DQB loci can be expressed (Ballingall et al. 2018b), including DQB2-like*01:01 which could not be recovered from cDNA here. It is possible that the alleles we were unable to retrieve from cDNA were not expressed or were expressed at undetectable levels within the peripheral blood samples tested or in the haplotypic combinations analysed, although it perhaps more likely reflects a combination of low cDNA quality and diversity in the primer binding sites. Whether the DQB2*09:01 allele is transcribed or not remains uncertain, but it cannot be ruled out that haplotype G carries three *DQB* loci. A previous study of MHC class IIa haplotypes using genomic DNA in Texel sheep identified a haplotype with three *DQA* and three *DQB* loci and another with two *DQA* and three *DQB* loci (Ali et al. 2016). Non-specific transcript analysis, such as RNAseq, would be valuable is assessing if all loci and alleles are transcribed. However, variation in the number of *DQ* loci and *DQ* haplotype configurations may be more common than previously thought in *O. aries*.

An unusual allele was identified on haplotype A which was identical to the *DQB-E1* sequence described by Herrmann-Hoessing (Unpublished, Accession number HQ728697.1) and described here as DQB2-like*03:01. This sequence, clustered with *DQB2-like* alleles, did not carry the single codon deletion within the second exon described in other *DQB2-like* alleles (Ballingall et al. 2018a, b) but does feature the typical *DQB2-like* 3’ UTR sequence (see Ballingall et al. 2018a). As haplotype A carries the *DQA2 + DQA2-like* configuration, it would be expected that it also carries the *DQB2 + DQB2-like* configuration. Allele DQB2-like*03:01 appears, therefore, to be a divergent *DQB2-like* allele. Recent developments in long read sequencing technologies, such as Oxford Nanopore or PacBio, would be valuable in determining which alleles are separate loci and which are divergent alleles, though high-quality, long-stranded DNA might be more easily obtained from domestic sheep not living on an isolated island.

Numerous alleles were shared among the MHC class IIa haplotypes within the Soay sheep. Pairwise alignment of the predicted amino acid sequences of second exons of the *DRB1*, *DQA* and *DQB* loci within each haplotype identified the greatest overall amino acid similarity between haplotypes A, G and A, E. However, the functional similarity of alleles is difficult to assess without detailed information on the diversity of peptides presented by each MHC molecule.

Variation in MHC class II DQ molecules is formed from the combination of α and β chains. *DQ* molecules are therefore the products of polymorphic *DQA1 + DQB1* and *DQA2 + DQB2* genes (Ballingall et al. 2018b). *DQA2-like + DQB2-like* combinations are likely to provide additional functional diversity as this combination has been shown to express at the cell surface following co-transfection (Ballingall et al. 2018b). Haplotypes E and G share the same DQA2*01:02:01 + DQA2-like*01:01:01 + DQB2-like*01:01:01 allelic combination, so both haplotypes will generate the same *DQ2-like* molecule. Therefore, an individual homozygous or heterozygous for haplotypes E and G might have fewer *DQ* molecules compared to other haplotype combinations. On the other hand, haplotype G carries two *DQB2* genes. If both are expressed and capable of forming functional molecules in combination with DQA2*01:02:01, it would have increased *DQ* molecule diversity. Intra-haplotype pairing of different *DQA* and *DQB* gene combinations may also provide additional class II molecules; however, not all allelic combinations are necessarily capable of generating functional molecules (Ballingall et al. 2018b).

The eight haplotypes characterised here are likely to be representative of all MHC class IIa variation in the Soay sheep. The *DRA* locus, which shows only limited allelic diversity in *O. aries* (Ballingall et al. 2010), was not genotyped here. It is unlikely that genotyping the *DRA* locus would have further subdivided any haplotypes. Whilst the haplotypes from only a small number of primarily homozygous animals were characterised, the haplotypes were validated at the *DRB1* and *DQA* loci in an additional 95 animals, and no new allelic combinations were identified. The *DQB* loci remain to be validated, and potential variation in the number of loci means that there is an unknown fraction of missing variation (Babik 2010). It is not certain that every haplotype has been detected; however, the extensive sequencing of multiple loci carried out throughout this study means that only alleles that occur at very low frequency will have been missed from our analysis.

Across all loci tested here, positive selection was found to be a more likely explanation for the observed diversity than neutrality, even comparing models M7 and M8 which are considered to be relatively robust to recombination (Anisimova et al. 2003). The strength of the signal was weakest at DQA2 (M2a and M8 *p* = 0.027), which is no longer significant after applying Bonferroni correction. Positively selected sites were also identified at all loci. Multiple PSS algorithms were tested due to their differing abilities to detect PSS under various selection scenarios, and as predicted, sites were not consistently detected by the three methodologies. The greatest variability was found at *DQA* and *DQB* (Fig. 2), which was not resolved by analysing the duplicated loci separately (Supplementary Fig. 4). This may be due to the complex nature of selection acting on the MHC, which could vary in space or time according to the parasite communities a population is exposed to, and therefore, different antigen-binding sites could be expected to experience different levels of continuous or episodic positive selection which are best detected using different algorithms. Recombination is unlikely to have a strong effect on PSS analyses (Anisimova et al. 2003). Nevertheless, there was a high level of congruence between PSS and antigen-binding sites in human homologues. This strongly suggests that there is a strong signal of positive selection within ovine MHC class IIa exon 2 loci, which is known to contain the antigen binding sites.

Whilst the *DRB1* locus remains the most polymorphic ovine MHC class II locus (106 *DRB1* alleles, compared to 27 *DQA* and 34 *DQB* alleles), by characterising the haplotypes within the Soay sheep, we are able to identify extensive variation at each of the *DQ* loci. The limited number of known alleles at the *DQ* loci compared to *DRB1* is due to the development of effective locus-specific primers for *DRB1* (Ballingall and Tassi 2010), whilst genotyping DQ allelic diversity has proved more challenging due to gene duplication and haplotype diversity. In contrast to the ovine DR molecule which is formed from the highly polymorphic *DRB1* and the relatively non-polymorphic *DRA* (Ballingall et al. 2010), at least two ovine DQ molecules are formed from combinations of the polymorphic and duplicated *DQA* and *DQB* loci (Scott et al. 1987; Wright and Ballingall 1994; Ballingall et al. 2015, 2018b). Additionally, diversity within the DQ molecules may be achieved by combining the products of two *DQA* loci with two *DQB* loci, although not all combinations were able to produce detectable MHC class II molecules on the surface of transfected cells (Ballingall et al. 2018b). The allelic diversity observed at the ovine DQ loci suggests that it is likely to be important in immune recognition of pathogens and the loci subject to corresponding selection pressures. Without haplotype characterisation, the full complement of MHC class IIa variation cannot be detected, which may limit the ability of subsequent evolutionary analyses to detect relationships between genotypes, phenotypes and fitness and therefore elucidate the evolutionarly mechanisms maintaining the high levels of diversity observed at the ovine MHC class IIa region.

Eight MHC class IIa haplotypes have been identified in the Soay sheep population through characterisation of allelic diversity at the *DRB1*, *DQA* and *DQB* genes. Additionally, PSS were identified at all three gene regions, which are likely to be involved in antigen-binding given their correspondence to antigen-binding sites in human and murine homologues. The diversity observed within the Soay sheep population is reduced compared to studies of domestic sheep (Ali et al. 2016; Koutsogiannouli et al. 2016), and, using this in-depth knowledge of the MHC class IIa haplotype diversity within the Soay sheep population, it should now be possible to develop a rapid SNP-based genotyping system in order to generate large-scale population data across the more than 30-year study of Soay sheep on St. Kilda. This would greatly facilitate analyses of evolutionary processes underlying the maintenance of the variation in the MHC class IIa region.

## Electronic supplementary material

ESM 1 (XLSX 20 kb)

Supplementary Figure 1. Frequency of DRB1 alleles in four cohorts of Soay sheep: 1993 (white), 1998 (light grey), 2003 (dark grey) and 2008 (black). (JPEG 52 kb)

Supplementary Figure 2. DQA exon 2 nucleotide sequences of alleles identified in Soay sheep. Nucleotides are numbered from the first base of the translation start codon in exon 1, and thus exon 2 begins at base 83. Dots (.) indicate identity to DQA1*03:01:01 and dashes (-) indicate missing sequence. (PNG 361 kb)

Supplementary Figure 3. DQB exon 2 nucleotide sequences of alleles identified in Soay sheep. Nucleotides are numbered from the first base of the translation start codon in exon 1, and thus exon 2 begins at base 110. Dots (.) indicate identity to DQB1*02:01:01 and dashes (-) indicate missing sequence. (PNG 409 kb)

Supplementary Figure 4. PSS for exon 2 of DQA and DQB loci analysed together and separately using BEB in CodeML, and FEL and MEME in HyPhy, where a coloured block indicates the codon was significant at >0.95 probability (CodeML) or p < 0.05 (HyPhy). Sites denoted * indicate this position was identified as an antigen biding site within the human orthologue (Brown et al. 1993; Stern and Wiley 1994; Reche and Reinherz 2003; Bondinas et al. 2007). (PNG 8 kb)
