# Supplementary figures and images for: Characterisation of major histocompatibility complex class IIa haplotypes in an island sheep population
Source: Immunogenetics. 2019 Feb 22;71(5):383–93. doi: 10.1007/s00251-019-01109-w (PMC6525122; doi:10.1007/s00251-019-01109-w)

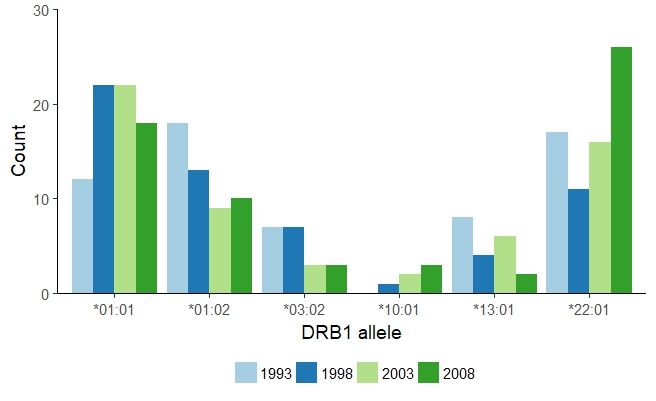

Supplement: Supplementary file 2 — Frequency of DRB1 alleles in four cohorts of Soay sheep: 1993 (white), 1998 (light grey), 2003 (dark grey) and 2008 (black). (JPEG 52 kb) [file 251_2019_1109_MOESM2_ESM.jpeg]

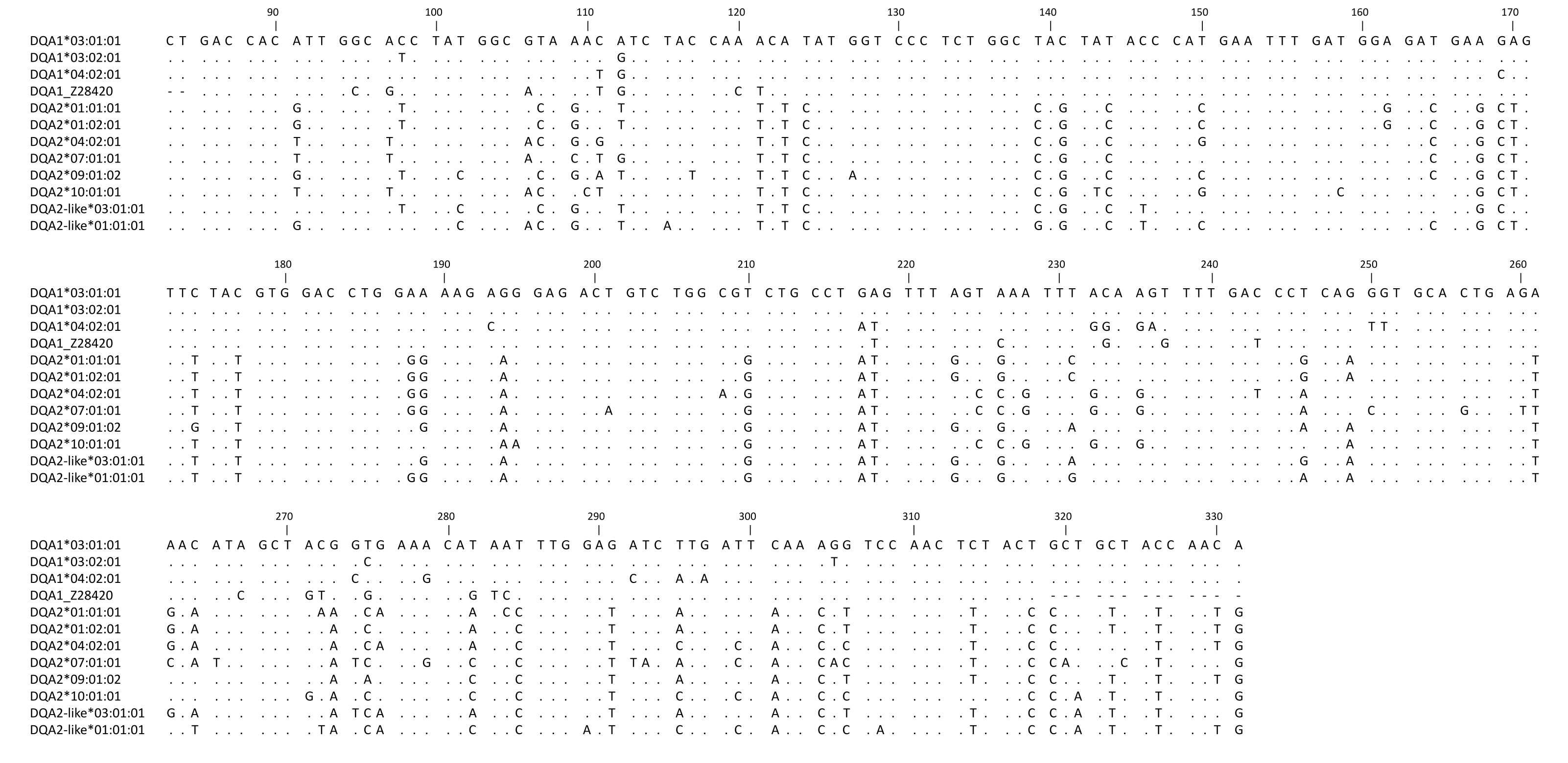

Supplement: Supplementary file 3 — DQA exon 2 nucleotide sequences of alleles identified in Soay sheep. Nucleotides are numbered from the first base of the translation start codon in exon 1, and thus exon 2 begins at base 83. Dots (.) indicate identity to DQA1*03:01:01 and dashes (-) indicate missing sequence. (PNG 361 kb) [file 251_2019_1109_MOESM3_ESM.png]

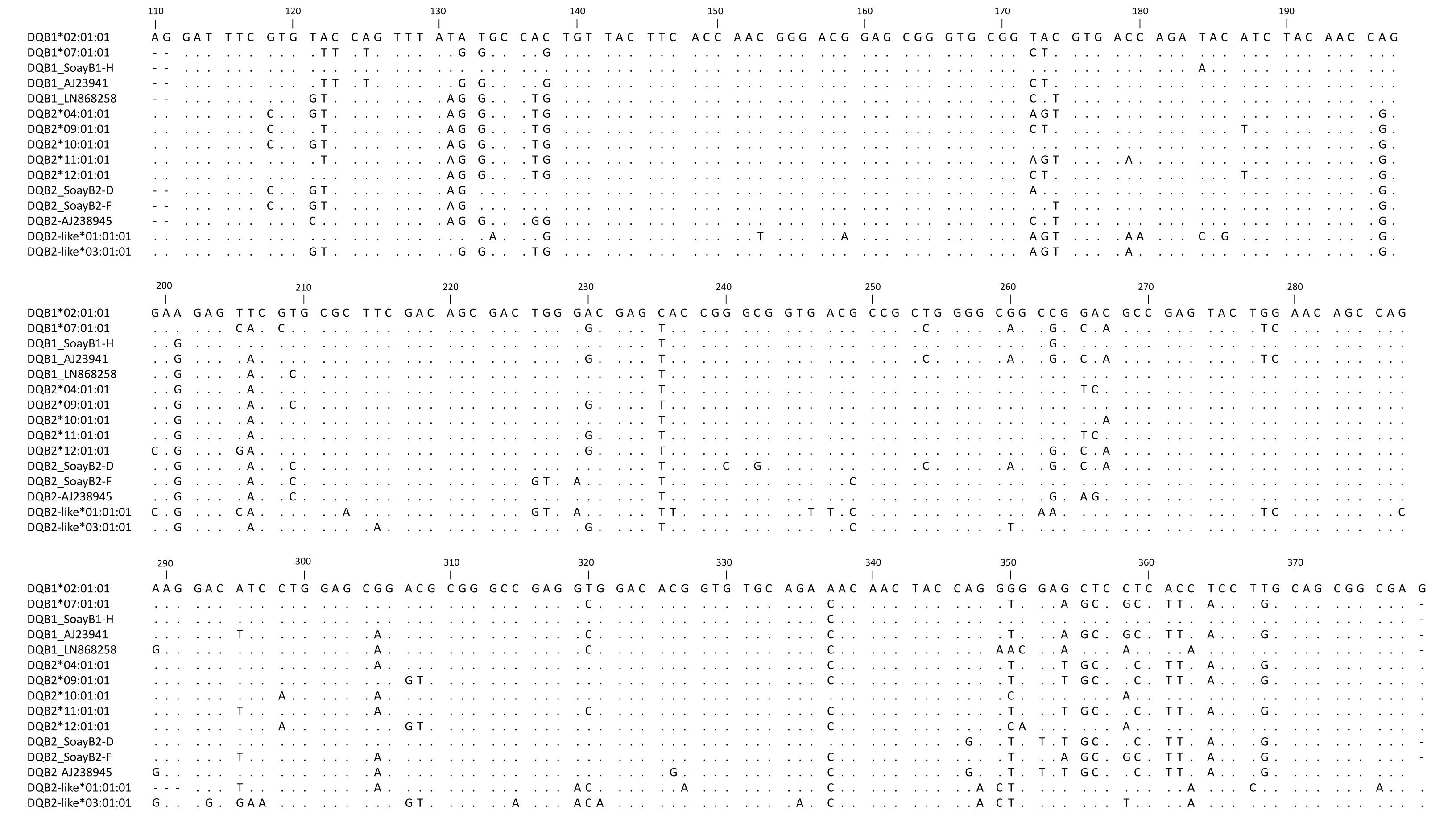

Supplement: Supplementary file 4 — DQB exon 2 nucleotide sequences of alleles identified in Soay sheep. Nucleotides are numbered from the first base of the translation start codon in exon 1, and thus exon 2 begins at base 110. Dots (.) indicate identity to DQB1*02:01:01 and dashes (-) indicate missing sequence. (PNG 409 kb) [file 251_2019_1109_MOESM4_ESM.png]

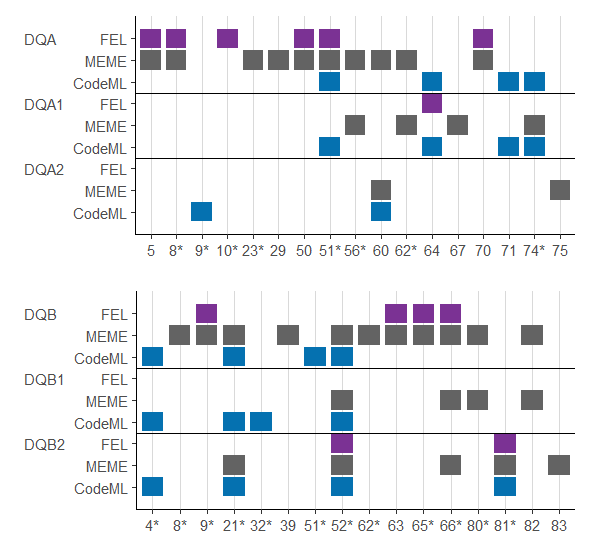

Supplement: Supplementary file 5 — PSS for exon 2 of DQA and DQB loci analysed together and separately using BEB in CodeML, and FEL and MEME in HyPhy, where a coloured block indicates the codon was significant at >0.95 probability (CodeML) or p < 0.05 (HyPhy). Sites denoted * indicate this position was identified as an antigen biding site within the human orthologue (Brown et al. 1993; Stern and Wiley 1994; Reche and Reinherz 2003; Bondinas et al. 2007). (PNG 8 kb) [file 251_2019_1109_MOESM5_ESM.png]
